# Identification of serum proteins AHSG, FGA and APOA-I as diagnostic biomarkers for gastric cancer

**DOI:** 10.1186/s12014-018-9194-0

**Published:** 2018-04-30

**Authors:** Feiyu Shi, Hong Wu, Kai Qu, Qi Sun, Fanni Li, Chengxin Shi, Yaguang Li, Xiaofan Xiong, Qian Qin, Tianyu Yu, Xin Jin, Liang Cheng, Qingxia Wei, Yingchao Li, Junjun She

**Affiliations:** 1 Department of General Surgery, The First Affiliated Hospital of Xi’an Jiao Tong University, 277 Yanta West Road, Xi’an, 710061 Shaanxi China; 2 Department of Hepatobiliary Surgery, The First Affiliated Hospital of Xi’an Jiao Tong University, 277 Yanta West Road, Xi’an, 710061 Shaanxi China; 3 Department of Talent Highland, The First Affiliated Hospital of Xi’an Jiao Tong University, 277 Yanta West Road, Xi’an, 710061 Shaanxi China; 4 Department of Cell Biology and Genetics, School of Basic Medical Sciences, Xi’an Jiao Tong University Health Science Center, 76 Yanta West Road, Xi’an, 710061 Shaanxi China; 5 Department of Developmental and Stem Cell Biology, Hospital for Sick Children, University of Toronto, Toronto, ON M5G0A4 Canada; 6 Department of Gastroenterology, The First Affiliated Hospital of Xi’an Jiao Tong University, 277 Yanta West Road, Xi’an, 710061 Shaanxi China

**Keywords:** Gastric cancer, Biomarker, APOA-I, AHSG, FGA

## Abstract

**Background:**

The development of clinically accessible biomarkers is critical for the early diagnosis of gastric cancer (GC) in patients. High-throughput proteomics techniques could not only effectively generate a serum peptide profile but also provide a new approach to identify potentially diagnostic and prognostic biomarkers for cancer patients.

**Methods:**

In this study, we aim to identify potentially discriminating serum biomarkers for GC. In the discovery cohort, we screened potential biomarkers using magnetic-bead-based purification and matrix-assisted laser desorption/ionization time-of-flight mass spectrometry in 64 samples from 32 GC patients that were taken both pre- and post-operatively and 30 healthy volunteers that served as controls. In the validation cohort, the expression patterns and diagnostic values of serum FGA, AHSG and APOA-I were further confirmed by ELISA in 42 paired GC patients (pre- and post-operative samples from 16 patients with pathologic stage I/II and 26 with stage III/IV), 30 colorectal cancer patients, 30 hepatocellular carcinoma patients, and 28 healthy volunteers.

**Results:**

ClinProTools software was used and annotated 107 peptides, 12 of which were differentially expressed among three groups (*P* < 0.0001, fold > 1.5). These 12 peptide peaks were further identified as FGA, AHSG, APOA-I, HBB, TXNRD1, GSPT2 and CAKP5. ELISA data suggested that the serum levels of FGA, AHSG and APOA-I in GC patients were significantly different compared with healthy controls and had favorable diagnostic values for GC patients. Moreover, we found that the serum levels of these three proteins were associated with TNM stages and could reflect tumor burden.

**Conclusion:**

Our findings suggested that FGA, AHSG and APOA-I might be potential serum biomarkers for GC diagnosis.

**Electronic supplementary material:**

The online version of this article (10.1186/s12014-018-9194-0) contains supplementary material, which is available to authorized users.

## Background

Gastric cancer (GC) is the fourth most common cancer with almost 1000,000 new cases diagnosed every year [1]. The incidence of GC is highest in Eastern Asia, especially in China, which alone accounts for nearly 50% of the world’s cases [2]. Moreover, GC is the second leading fatal cancer subtype, and approximately 498,000 Chinese patients died from GC in 2015 [3]. The high mortality rate of GC is mainly due to delayed diagnosis, at which time the cancer has advanced to an inoperable stage and can no longer be eradicated by surgical resection [4]. There are non-specific symptoms displayed in GC patients at the early stages [5]. Therefore, exploring novel biomarkers for GC patients will help monitor tumor status and guide clinical treatment.

Serum tumor biomarkers can be secreted by tumor cells or by normal cells responding to the malignant behavior of tumors [6]. For decades, serum-based biomarkers were considered the most important biomarkers to reflect tumor burden and have been applied for cancer diagnosis and post-operation monitoring. The conventional serum-based biomarkers for GC, such as carcino-embryonic antigen (CEA), carbohydrate antigen 19-9 (CA19-9) and CA72-4, did not have favorable specificity and sensitivity, which always resulted in delayed diagnosis [7, 8]. Ebert MP et al. stated that the sensitivities of above three biomarkers are only 16–63, 20–56, and 18–51%, respectively [9].

In recent years, several high-throughput proteomics techniques have been applied in serum samples to uncover novel diagnostic markers [10]. Matrix-assisted laser desorption/ionization time-of-flight mass spectrometry (MALDI-TOF–MS) is becoming a standard tool in protein analysis in particular [11–13]. In this study, we first evaluated a discovery group that included 32 GC patients and 30 healthy volunteers and employed MALDI-TOF–MS to identify peptides that were candidate biomarkers for GC. Next, we evaluated a validation group that included 42 paired GC patients, 30 colorectal cancer (CRC) patients, 30 hepatocellular carcinoma (HCC) patients, and 28 healthy volunteers and performed enzyme-linked immunosorbent assay (ELISA) to validate the diagnostic values of the candidate biomarkers identified in the first step.

## Methods

### Patient selection and sample preparation

The research protocol was approved by the Ethics and the Human Research Review Committee of Xi’an Jiao Tong University. All subjects signed a consent form before participating in this research study, which was approved by the Institutional Review Board of Xi’an Jiao Tong University. All experiments were carried out in accordance with the approved guidelines. A total of 266 serum samples from 192 individuals were collected from the Department of General Surgery, Department of Gastroenterology, the Department of Physical Examination, the First Affiliated Hospital of Xi’an Jiao Tong University, China, from March 2016 to April 2017. The discovery cohort consisted of 32 pairs of serum samples from 32 pre- and post-operative GC patients as well as 30 healthy controls. The validation cohort was composed of 42 pairs of serum samples from GC patients (16 pairs are at I/II stage, and 26 pairs are at III/IV stage), 30 CRC patients, 30 HCC patients, and 28 healthy volunteers. The diagnosis of GC, CRC and HCC was confirmed by pathological diagnosis. The discovery cohort and validation cohort are completely non-overlapping. Moreover, the healthy control groups were gender- and age-matched with the cancer groups. The characteristic information of all subjects is shown in Table 1.

**Table 1:** Demographics of all subjects enroll in this study

Patients characteristics	Discovery cohort		Validation cohort			
	Control group	GC	Control group	GC	CRC	HCC
Number of cases	30	32	28	42	30	30
Gender						
Male/female	20/10	22/10	18/10	28/14	21/11	22/8
Age (year)	65.44 ± 7.85	63.97 ± 7.42	62.48 ± 8.68	63.38 ± 9.35	61.80 ± 9.42	60.73 ± 9.28
pTNM stage						
I	–	6	–	5	6	4
II	–	10	–	11	10	9
III	–	12	–	21	11	14
IV	–	2	–	5	3	3

The exclusion criteria for subjects were as follows: (1) patients with a known history of any other tumors and any obvious inflammatory diseases, such as liver cirrhosis, chronic renal disease, and diabetes mellitus; (2) patients with a known history of any surgical operations, chemotherapy or radiotherapy before collection of the serum; and (3) patients with a known history of receiving blood transfusion within a month before collection of the serum.

All blood samples were obtained from non-fasting patients or healthy controls in the morning. The serum samples were collected in 10-cc separator tubes (BD, #367820) and were kept at 4 °C for 1 h, then centrifuged at 3000 rpm for 10 min at 4 °C. The serum samples were distributed into 400-μL aliquots and stored at − 80 °C until use.

### MS analysis: magnetic beads-based immobilized metal-ion affinity chromatography (MB-IMAC-Cu) fractionation and MALDI-TOF–MS

Magnetic Beads-based Immobilized Metal-ion Affinity Chromatography (MB-IMAC-Cu) (ClinProt purification reagent sets; Bruker Daltonics, Bremen, Germany) was used for enrichment of serum peptides followed by MALDI-TOF–MS analysis. A total of 94 serum samples were fractionated according to instructions provided by Bruker Daltonics. Briefly, 5 μl of magnetic beads was pretreated with 50 μl of binding buffer, and the supernatant was carefully discarded. The magnetic beads were re-suspended in 20 μl of binding buffer in a PCR tube, and then 5 μl of serum sample was added and mixed gently. The mixtures were incubated at room temperature for 5 min and separated in the magnetic separator. The beads were washed once with 100 μl of wash buffer, and the peptides and proteins were eluted with 10 μl of elution buffer from beads. Then, 1 μl of the eluted peptides and proteins and 1 μl of a mixture containing 3 mg/ml α-cyano-4-hydroxy-cinnamic acid (Bruker) in 50% acetonitrile and 0.5% trifluoroacetic acid was spotted onto the MALDI AnchorChip surface. Samples were spotted in triplicate to evaluate the reproducibility of this method.

### Data analysis with ClinProTools

Air-dried targets were immediately tested with calibrated Autoflex III MALDI-TOF–MS (Bruker), flexControl version 3.0 software (Bruker), via an optimized measuring protocol. The settings of the instrument were as follows: ion source 1, 20.00 kV; ion source 2, 18.90 kV; lens, 6.50 kV; and pulsed ion extraction, 120 ns. Ionization was achieved by irradiation with a crystal laser operating at 200.0 Hz. A standard calibration mixture of peptides and proteins (mass range 1–10 kDa) was used for mass calibration. For each MALDI spot, 1200 spectra were acquired (200 laser shots at 6 different spot positions). All tests were performed in a blinded manner, including the serum analysis of different groups. The Flex analysis software (version 3.0; Bruker) was applied for all serum data analysis. Recognition of peptide patterns was analyzed by ClinProTools version 2.2 software (Bruker).

### Peptide identification by LC–ESI–MS/MS

After completing the statistical analysis, the peptides were identified using liquid chromatography-mass spectrometry, which combined Nano Acquity UPLC liquid chromatography (Waters, USA) with an LTQ Orbitrap XL mass spectrometer system (Thermo Fisher Scientific, USA). The Peptide mixture solutions purified by MB-IMAC-Cu trapping used a captrap C18 (2 mm × 0.5 mm) column (Michrom Corporation, USA) and an analytical Magic C18, AQ (100 µm × 150 mm) column (Michrom Corporation). Mobile phase A was a solution of 5% acetonitrile and 0.1% formic acid, and mobile phase B was a solution of 90% acetonitrile and 0.1% formic acid. Peptide mixtures were injected into the trap column with a flow of 20 μl/min for 5 min and then eluted with a three-step linear gradient, starting from 5% B to 45% B for 40 min, increased to 80% B for 1 min, and then held at 80% B for 4 min. The column was re-equilibrated at the initial conditions for 15 min. The column flow rate was maintained at 500 nl/min, and the column temperature was maintained at 35°C.

Electrospray voltage of 1.9 kV versus the inlet of the mass spectrometer was used. The LTQ Orbitrap XL mass spectrometer was operated in the data-dependent mode to switch automatically between MS and MS/MS acquisition. Survey full scan MS spectra with two microscans (m/z 400–2000) were acquired in the Obitrap with a mass resolution of 100,000 at m/z 400, followed by eight sequential LTQ-MS/MS scans. Dynamic exclusion was used with two repeat counts, consisting of a 10 s repeat duration and a 60 s exclusion duration. For MS/MS, precursor ions were activated using 25% normalized collision energy at the default activation q of 0.25. All MS/MS spectra were profiled with SEQUEST [v.28 (revision 12), Thermo Electron Corp.] which searched the human International Protein index (IPI) database (IPI human v3.64 fasta with 71,983 entries) and the UniprotKB (http://www.uniprot.org) for peptide-to-spectral matching. To minimize false positives, a decoy database containing all of the reverse protein sequences was added to this database. The search parameters were as follows: no enzyme digestion, the variable modification was the oxidation of methionine, a peptide mass tolerance of 20 ppm, and a fragment ion tolerance of 1.0 Da.

The resulting filter parameters were as follows: ^∆^Cn ≥ 0.10, Xcorr ≥ 2.3 for two charged ions, Xcorr ≥ 2.6 for three charged ions, Xcorr ≥ 3.0 for four or more charged state ions, and FDR < 0.01. The errors were less than 0.1 Da in the m/z of peptide determined by LC–MS.

### Enzyme-linked immunosorbent assay (ELISA)

All serum samples were run in triplicate and analyzed in a blinded fashion in triplicate. The concentrations of Isoform I of Fibrinogen alpha chain precursor (FGA), Alpha-2-HS-glycoprotein precursor (AHSG) and Apolipoprotein A-I precursor (APOA1) were quantified with a Human FGA ELISA Kit (Elisa Biotech, #: CK-E93791H), a Human AHSG ELISA Kit (Elisa Biotech, #: CK-E95306H), and a Human ApoA-1 ELISA kit (Elisa Biotech, #: CK-E11517H), respectively. Standard curves were generated and used to determine the concentrations of FGA, AHSG and APOA-1 in the samples analyzed.

### Statistical analysis

Statistical analysis was performed with GraphPad Prism version 6.0 (GraphPad Software, La Jolla, CA, USA). All data are shown as the mean ± SD. *P* < 0.05 was considered significant. Comparisons among multiple groups were performed via repeated measures analysis of variance and the least significant difference test. Student’s t test was used to analyze the ELISA data. ROC curves were utilized to assess the diagnostic value of FGA, AHSG, and APOA-I.

## Results

### Serum proteomic profiling of GC patients and healthy controls

**Fig. 1:**
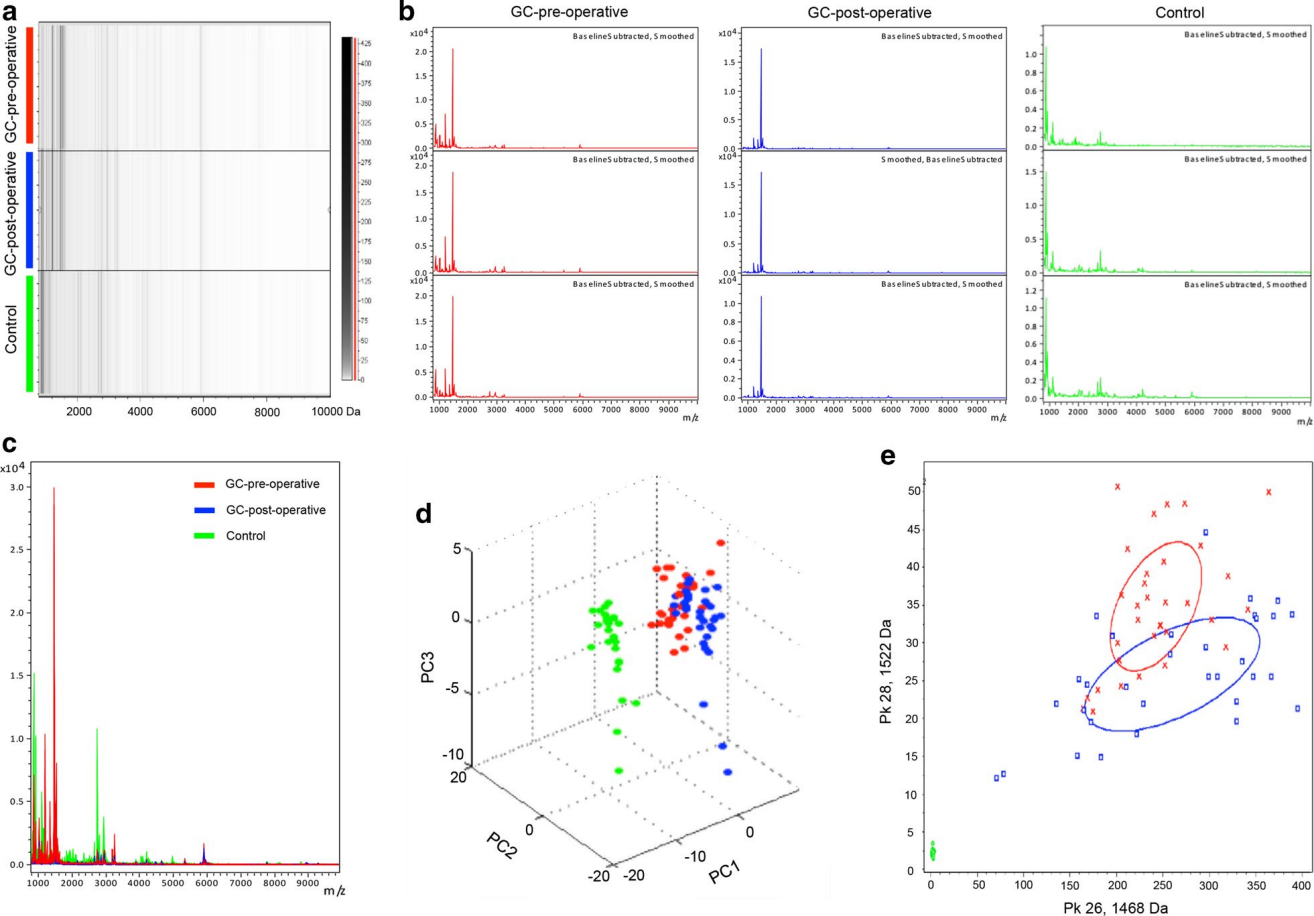
Reproducibility of mass spectra generated in individuals from different groups and the comparative analysis of serum proteomic profiling between different groups. **a** Gel view of mass spectra from healthy control (green), GC pre-operative (red) and GC post-operative (blue) serum samples, in the mass range from 1000 to 10000 Da. **b** Representative mass spectra of three samples in each group in the mass range from 1000 to 10,000 Da, showing high reproducibility and stability between replicates. **c** Overall sum of the spectra in the mass range from 1000 to 10,000 Da obtained from all GC patients pre-operation (red) and post-operation (blue), as well as healthy controls (green). **d** 3D plot and bivariate plot **e** of pre-operative GC patients (red), healthy controls (green), and post-operative GC patients (blue) in the PCA

As shown in Fig. 1b, the reproducibility and stability of the mass spectra data were closely reproducible in triplicate samples of each group. We then performed mass spectrometry-based proteomics via the MALDI-TOF–MS method (Fig. 1a). Our data revealed that the mass spectra differed among the pre-operative GC patients (red), post-operative GC patients (blue) and healthy controls (green) (Fig. 1). Additionally, the serum samples fractionated by MB-IMAC-Cu and MALDI-TOF–MS showed that pre-operative GC patients (red), post-operative GC patients (blue) and controls (green) had proteomic profiles that ranged from 1000 to 10,000 Da (Fig. 1c). Principal component analysis revealed that pre-operative GC patients (red), post-operative GC patients (blue) and control (green) samples could be distinguished by several peptides (Fig. 1d, e), which suggested the possibility of exploring serum biomarkers to separate GC patients from control subjects.

### Selection of differential expressed peptides and diagnostic model testing

**Fig. 2:**
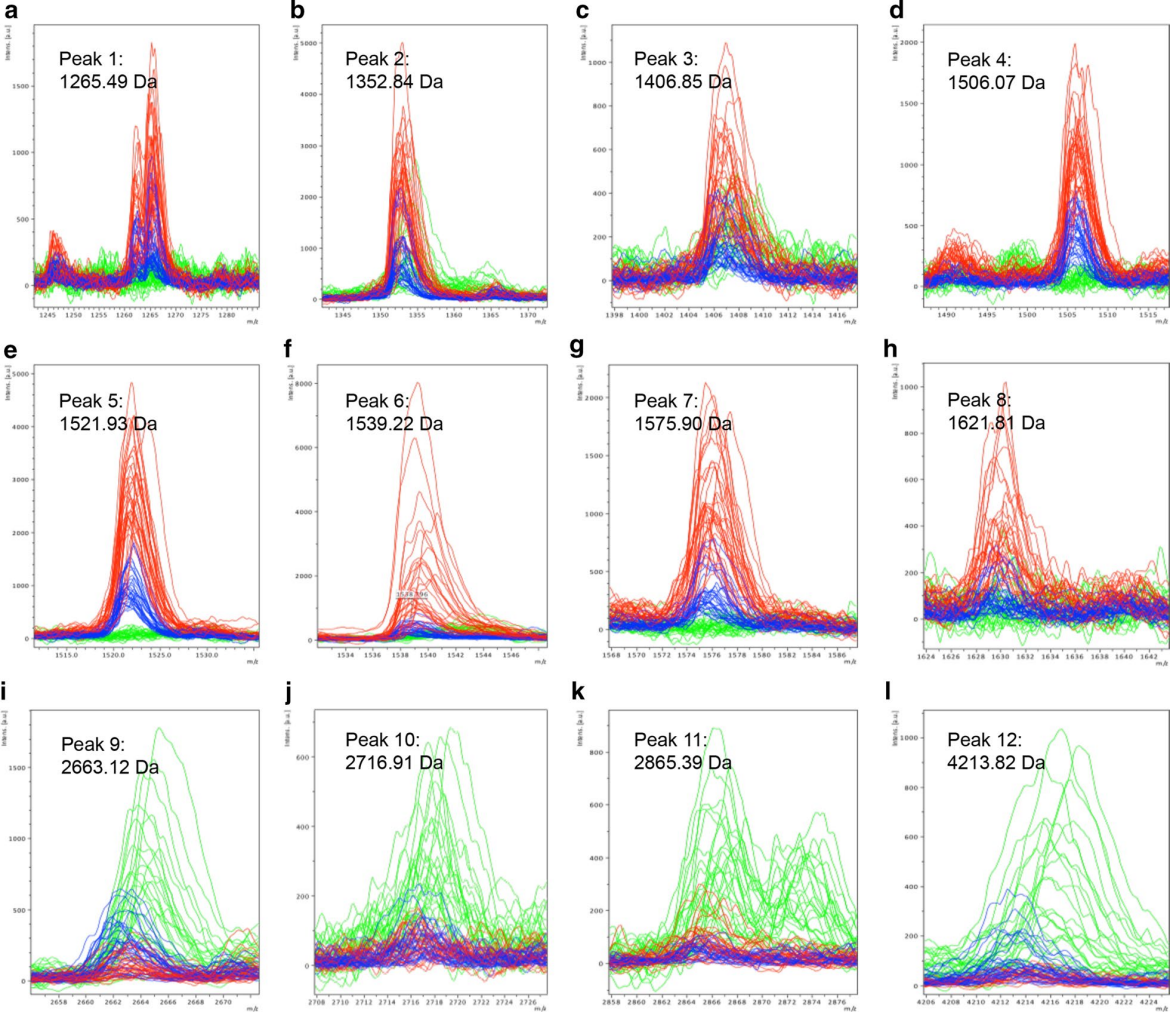
Comparison of the spectra of the 12 potential peptides in three different groups (red: pre-operative GC patients, blue: post-operative GC patients, green: healthy controls). **a** Peak 1, m/z: 1265.49 Da; **b** peak 2, m/z: 1352.84 Da; **c** peak 3, m/z: 1406.85 Da; **d** peak 4, m/z: 1506.07 Da; **e** peak 5, m/z: 1521.93 Da; **f** peak 6, m/z: 1539.22 Da; **g** peak 7, m/z: 1575.90 Da; **h** peak 8, m/z: 1621.81 Da; **i** peak 9, m/z: 2663.12 Da; **j** peak 10, m/z: 2716.91 Da; **k** peak 11, m/z: 2865.39 Da; and **l** peak 12, m/z: 4213.82 Da

**Fig. 3:**
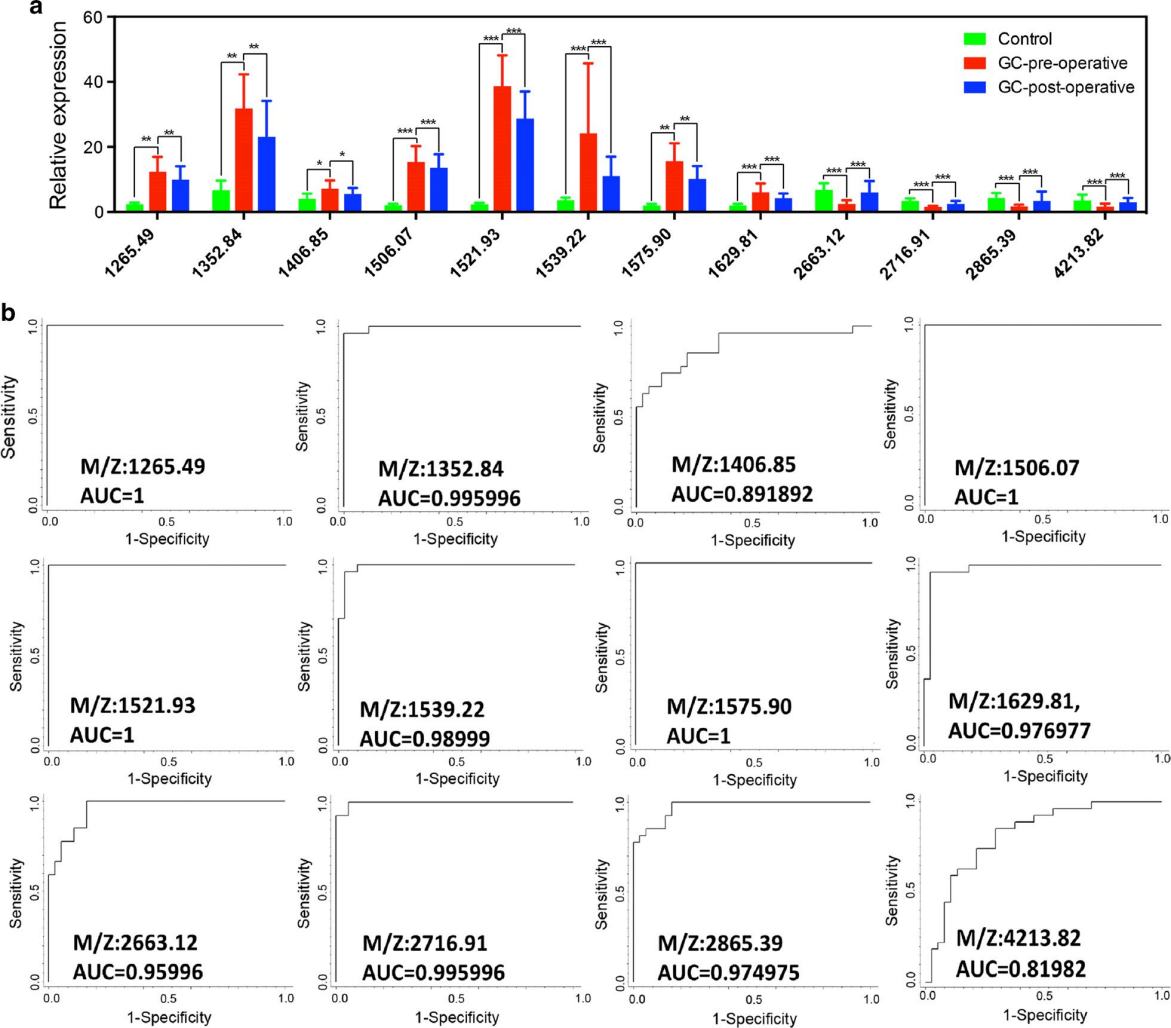
The expression patterns and diagnostic accuracies of 12 peptides in GC patients. **a** Comparison of the expression levels of the 12 peptides in three different groups. ***P < 0.0001; **P < 0.001; *P < 0.05; **b** The ROC curves and AUC values of 12 peptides in GC patients and healthy controls

ClinProTools software (version 2.2, Bruker) was used to identify a total of 107 different peaks from serum samples. Among them, 12 peaks were significantly different among pre-operative and post-operative GC patients and healthy controls (*P *< 0.0001, fold change > 1.5) (Fig. 2) and showed a response to therapy and a tendency to return to healthy control values after the operation. Peptide peaks 1–8 (peak 1, m/z: 1265.49 Da; peak 2, m/z: 1352.84 Da; peak 3, m/z: 1406.85 Da; peak 4, m/z: 1506.07 Da; peak 5, m/z: 1521.93 Da; peak 6, m/z: 1539.22 Da; peak 7, m/z: 1575.90 Da; and peak 8, m/z: 1621.81 Da) were up-regulated, and peaks 9–12 (peak 9, m/z: 2663.12 Da; peak 10, m/z: 2716.91 Da; peak 11, m/z: 2865.39 Da; and peak 12, m/z: 4213.82 Da) were down-regulated in pre-operative GC patients compared with healthy controls (Table 2). The relative expression levels of the above 12 peptide peaks among healthy controls (green), pre-operative GC (red), and post-operative GC (blue) are shown in Fig. 3a. The receiver operating characteristic (ROC) curves of 12 differentially expressed peptides between GC patients and healthy controls are shown in Fig. 3b. The area under ROC (AUC) values of all peptides were more than 0.8, suggesting these peptides might be potential biomarkers for GC.

**Table 2:** Mean levels of different peptides among healthy controls, pre-operative GC patients and post-operative GC patients

Peaks	Mass (Da)	*P* value	Control	Pre-	Post-
1	1265.49↑	0.000145	2.44 ± 0.5	12.34 ± 4.66	9.93 ± 4.13
2	1352.84↑	0.000111	6.73 ± 2.91	31.8 ± 10.48	23.07 ± 11.08
3	1406.85↑	0.00222	3.98 ± 1.68	7.2 ± 2.49	5.57 ± 1.86
4	1506.07↑	0.00000893	2.09 ± 0.46	15.42 ± 4.85	11.60 ± 3.14
5	1521.93↑	< 0.000001	2.41 ± 0.43	38.66 ± 9.47	28.69 ± 8.36
6	1539.22↑	< 0.000001	3.71 ± 0.78	24.13 ± 10.59	11.03 ± 6.02
7	1575.90↑	0.000251	2.02 ± 0.44	15.66 ± 5.49	10.17 ± 3.96
8	1629.81↑	< 0.000001	2.02 ± 0.53	6.13 ± 2.7	4.24 ± 1.51
9	2663.12↓	0.0000226	6.81 ± 2.05	2.53 ± 1.12	6.07 ± 3.45
10	2716.91↓	0.0000935	3.46 ± 0.72	1.55 ± 0.4	2.53 ± 0.9
11	2865.39↓	< 0.000001	4.3 ± 1.54	1.71 ± 0.58	3.42 ± 2.87
12	4213.82↓	< 0.000001	3.64 ± 0.73	1.61 ± 0.98	2.96 ± 1.36

### Identification of selected serum peptides in GC patients

All 12 peptide peaks were further identified using LC–ESI–MS/MS and human International Protein Index (IPI) database and UniprotKB. The results of peptide fragmentation identification are shown in Table 3. The peaks at m/z 1265.49 Da, 1352.84 Da, 1575.90 Da, 2663.12 Da were identified as Isoform I of Fibrinogen alpha chain precursor (FGA). The peaks at m/z 1506.07 Da, m/z 1521.93 Da, m/z 1629.81 Da, m/z 2716.91 Da, m/z 2865.39 Da and m/z 4213.82 Da were identified as Alpha-2-HS-glycoprotein precursor (AHSG), Apolipoprotein A-I precursor (APOA-I), Hemoglobin subunit beta (HBB), Isoform 5 of Thioredoxin reductase 1, cytoplasmic (TXNRD1), Cytoskeleton-associated protein 5 (CKAP5) and Eukaryotic peptide chain release factor GTP-binding subunit ERF3B (GSPT2), respectively. The relevant peptide sequences are listed in Table 3. There is no matching information for peptide fragmentation identification of the peak at m/z 1406.85 and m/z 1539.22 in the above databases. The results of identification peptide are shown in Additional file 1.

**Table 3:** Sequence identification of six potential GC biomarkers

Mass (m/z)	Peptides sequence	International Protein Index	Identity
1521.93 Da	L.SALEEYTKKLNTQ. -	IPI:IPI00021841.1	Apolipoprotein A-I precursor (APOP-I)
1265.49 Da 1352.84 Da 1575.90 Da 2663.12 Da	S.GEGDFLAEGGGVR.G D.SGEGDFLAEGGGVR.G Q.FTSSTSYNRGDSTF.E A.DEAGSEADHEGTHSTKRGHAKSRPV.R	IPI:IPI00021885.1	Isoform I of Fibrinogen alpha chain precursor (FGA)
2716.91 Da	R.VVAQSTNSEEIIEGEYNTVMLAIGR.D	IPI:IPI00554786.5	Isoform 5 of Thioredoxin reductase 1, cytoplasmic (TXNRD1)
4213.82 Da	K.EQSDFCPWYTGLPFIPYLDNL PNFNRSIDGPIRLPI.V	IPI:IPI00642097.1	Eukaryotic peptide chain release factor GTP-binding subunit ERF3B (GSPT2)
1506.07 Da	G.VVSLGSPSGEVSHPR.K	IPI:IPI00022431.1	Alpha-2-HS-glycoprotein precursor (AHSG)
1629.81 Da	F.GDLSTPDAVMGNPKVK.A	IPI:IPI00654755.3	Hemoglobin subunit beta (HBB)
2865.39 Da	L.DKIKECSEKVELIHGKKAGLAADKKE.F	IPI:IPI00028275.1	Cytoskeleton-associated protein 5 (CKAP5)

### Validation of expression patterns for three biomarkers in cancers by ELISA

**Fig. 4:**
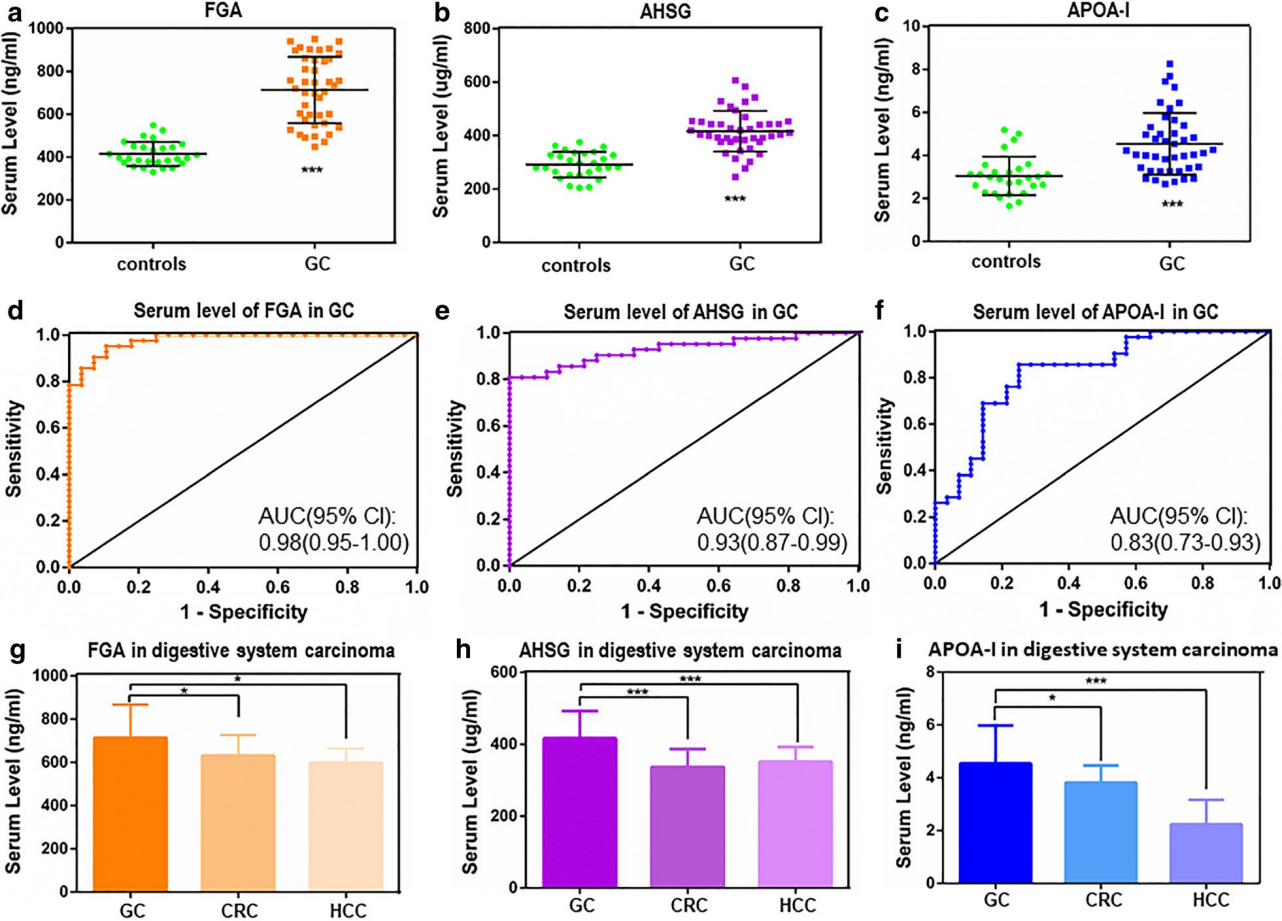
The expression patterns, diagnostic accuracies and specificity of three proteins. The expression patterns of FGA (**a**), AHSG (**b**) and APOA-I (**c**) in GC patients. ***P < 0.0001 versus controls; **P < 0.001 versus controls; *P < 0.05 versus controls. The diagnostic accuracies of FGA (**d**), AHSG (**e**) and APOA-I (**f**) in GC patients. The specificity of FGA (**g**), AHSG (**h**) and APOA-I (**i**) in three common digestive system carcinomas, ***P < 0.0001 versus GC; **P < 0.001 versus GC; *P < 0.05 versus GC

To validate the selected serum proteins that were identified by proteomics, a validation cohort of 42 GC patients, 30 CRC patients, 30 HCC patients and 28 healthy controls was evaluated in the present study. First, we chose three proteins, FGA, AHSG and APOA-I, according to the relative expression levels of the 12 peptides in three different groups and validated in GC by ELISA (Table 4). Our results revealed that the serum FGA levels were significantly higher in GC patients than in controls, and the ROC analysis showed high diagnostic values of serum FGA in GC with AUCs of 0.98 (Fig. 4a, d). In addition, the serum AHSG and APOA-I levels were noticeably elevated in GC patients compared with controls. Moreover, the ROC analysis also demonstrated high diagnostic values of serum AHSG and APOA-I in GC with AUCs of 0.92 and 0.83, respectively (Fig. 4b, c, e, f). Finally, to further determine the specificity of FGA, AHSG and APOA-I in GC patients, we examined the serum levels in patients with three common digestive carcinomas (CRC, HCC, and above GC) by ELISA. These three candidates had higher serum levels in GC than in CRC and HCC (*P* < 0.05, both) (Fig. 4g, h, i). All results indicated that FGA, AHSG and APOA-I could be considered valuable diagnostic biomarkers for GC.

**Table 4:** The serum expression level of FGA, APOA-1 and AHSG in different groups

	Control group (n = 28)	I/II GC-pre (n = 16)	III/IV GC-pre (n = 26)	I/II GC-post (n = 16)	III/IV GC-post (n = 26)	CRC (n = 30)	HCC (n = 30)
FGA (ng/ml)	406.80 ± 42.52 (330.14–490.51)	544.05 ± 53.74 (447.79–642.26)	814.19 ± 85.74 (676.96–950.09)	403.54 ± 46.80 (428.47–598.98)	664.13 ± 63.38 (556.04–755.03)	630.94 ± 96.46 (429.35–800.72)	596.70 ± 67.63 (450.21–779.29)
AHSG (μg/ml)	291.83 ± 47.24 (204.48–375.66)	353.74 ± 49.51 (244.96–441.11)	455.79 ± 59.74 (385.38–606.92)	324.74 ± 41.81 (244.41–401.99)	420.17 ± 53.98 (354.95–548.11)	337.34 ± 49.78 (224.17–423.79)	351.26 ± 41.66 (262.71–441.91)
AOPA-I (μg/ml)	3.44 ± 0.96 (2.03–5.44)	5.91 ± 1.19 (4.66–8.23)	3.71 ± 0.79 (2.67–6.19)	4.52 ± 1.14 (3.21–6.92)	3.31 ± 0.82 (1.97–5.02)	3.82 ± 0.65 (2.80–5.67)	2.25 ± 0.93 (0.84–4.10)

### Diagnostic values of three biomarkers in GC patients with early or late stage

**Fig. 5:**
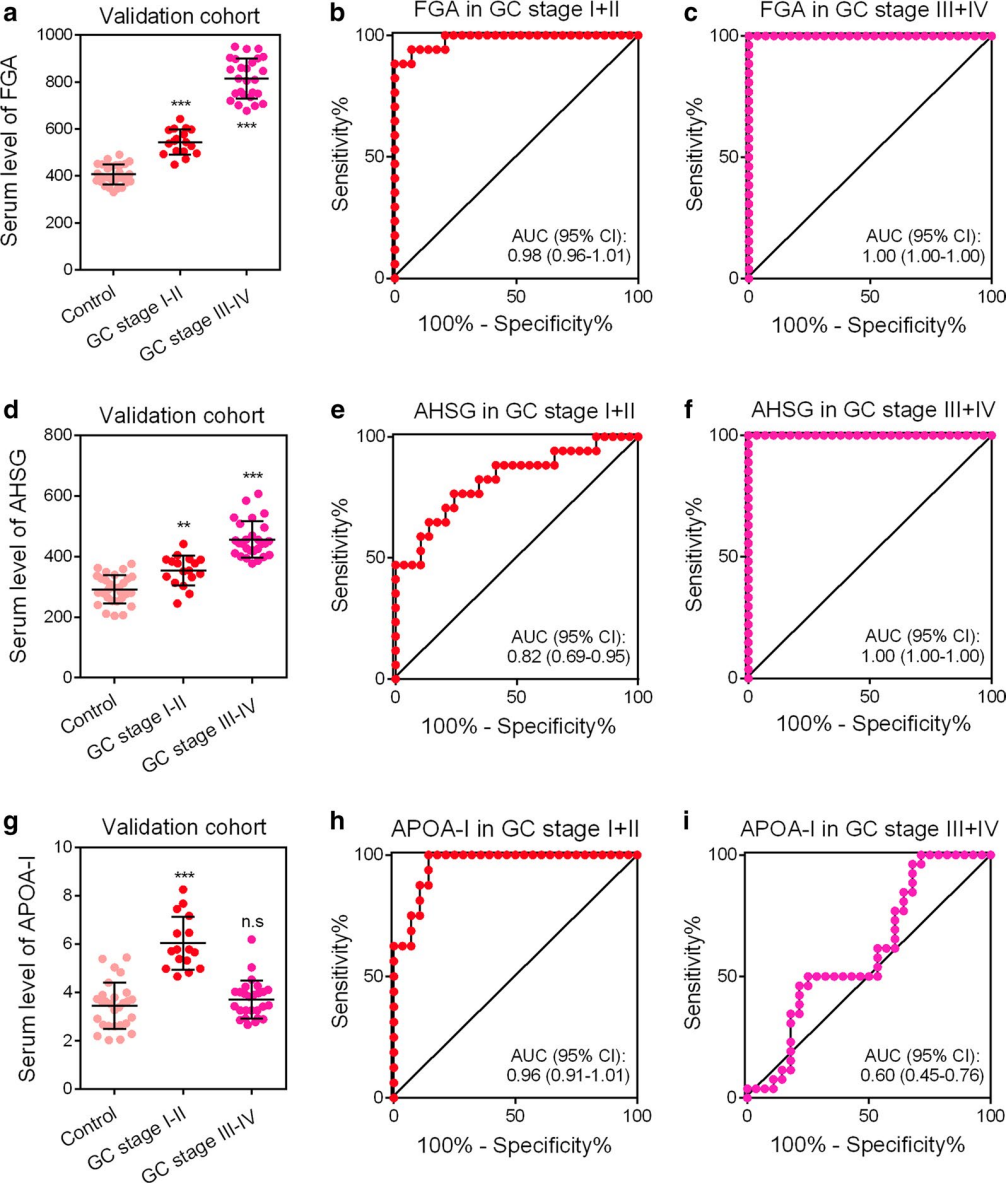
The diagnostic accuracies of three proteins in patients with different stages of GC. The diagnostic accuracies of FGA (**a**), AHSG (**d**) and APOA-I (**g**) in GC patients. The diagnostic accuracies of FGA (**b**), AHSG (**e**) and APOA-I (**h**) in GC patients with stage I + II GC. The diagnostic accuracies of FGA (**c**), AHSG (**f**) and APOA-I (**i**) in GC patients with stage III + IV GC. ***P < 0.0001 versus controls; **P < 0.001 versus controls; *P < 0.05 versus controls

**Fig. 6:**
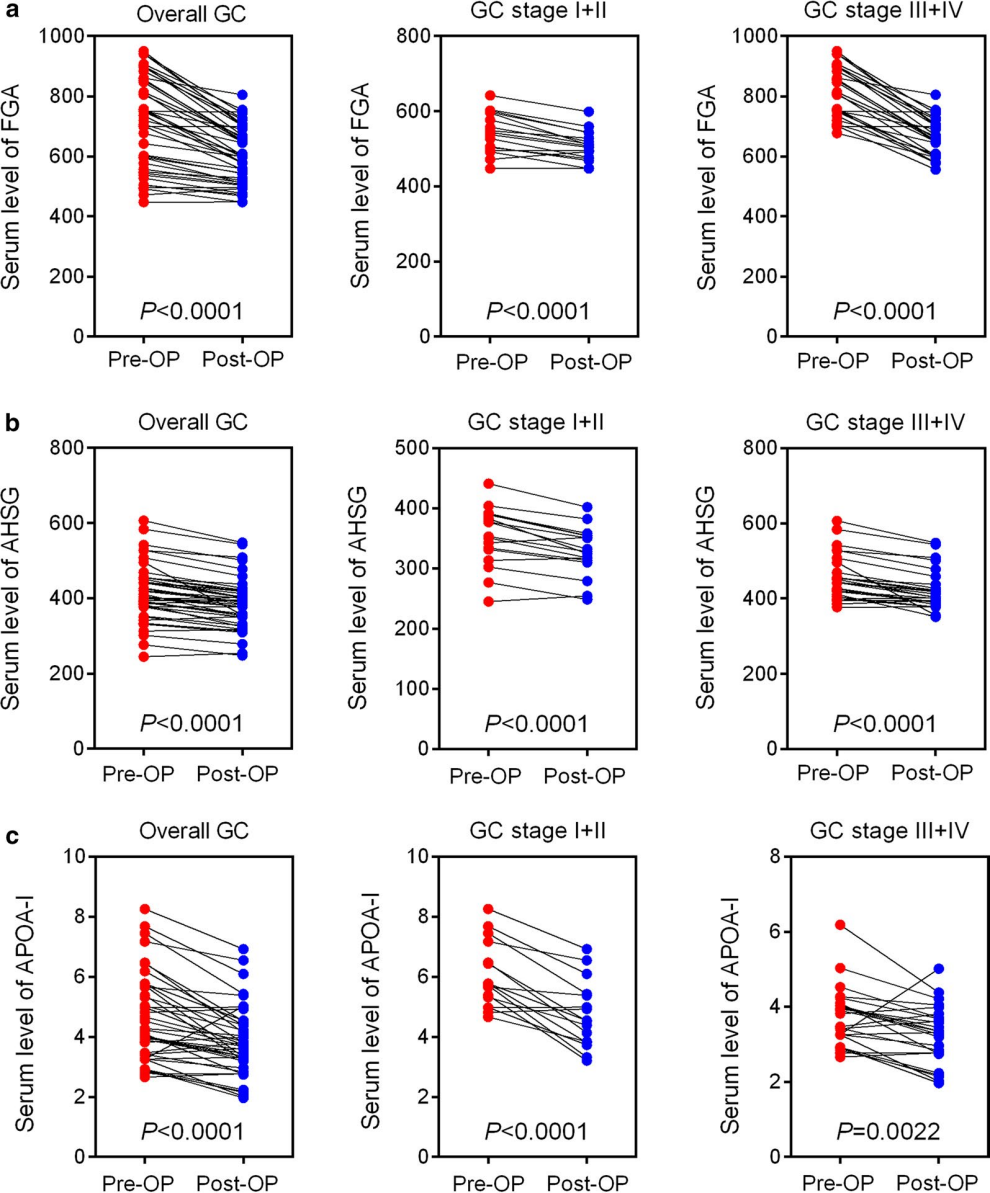
Comparison of three protein levels between pre- and post-operative groups. **a** The expression level of FGA in pre- and post-operative groups. **b** The expression level of AHSG in pre- and post-operative groups. **c** The expression level of APOA-I in pre- and post-operative groups

We also evaluated the expression patterns of three biomarkers in patients with early- or late-stage GC. A total of 16 patients with early-stage GC (pTNM I + II), 26 patients with late-stage GC (pTNM III + IV), and 28 healthy controls were included. Our results showed that the serum level of FGA was elevated along with GC progression (Fig. 5a). Serum FGA showed favorable diagnostic values for GC patients both in the early stage (Fig. 5b) and late stage (Fig. 5c). Similarly, the serum level of AHSG was also elevated with an increase in tumor stage (Fig. 5d). The diagnostic value of serum AHSG in GC patients with late-stage GC (Fig. 5f) was significantly higher than in patients with early-stage GC (Fig. 5e). In contrast, the serum level of APOA-I was only elevated in GC patients with early-stage GC (Fig. 5g). The diagnostic value of serum APOA-I was significantly higher in patients (Fig. 5h) with early-stage GC than those with late-stage GC (Fig. 5i). Furthermore, we also compared the expression levels of three biomarkers between pre- and post-operation groups. Our results showed that the serum levels of three biomarkers were all decreased after the operation, suggesting that those biomarkers could reflect tumor burden (Fig. 6).

## Discussion

GC is a highly aggressive cancer associated with high mortality in China. Due to the lack of early detection, most GC patients are diagnosed with advanced-stage disease, for which treatment options are limited, resulting in an overall 5-year survival rate of 10–28% [14]. Serum-based biomarkers are of considerable importance in the early diagnosis of various diseases including cancer [10]. Due to the complex mixture in serum samples, the identification of potential tumor biomarkers secreted in very tiny amounts is very difficult [15, 16]. The development of new proteomics techniques has greatly advanced in this field [17]. The enrichment of protein from serum can be screened with MB-IMAC-Cu, which is designed for extremely sophisticated biomarker profiling studies on a discovery level. Magnetic bead-based fractionation followed by MALDI-TOF–MS combined with advanced bio-informatic tools (ClinProTools software) can identify biomarkers effectively and precisely [18]. In the current study, we employed MALDI-TOF/MS to screen candidate peptides from serum samples. A total of 12 candidate peptides were selected and then identified as the fragment of FGA, AHSG, APOA-I, HBB, CKAP5 and GSPT2 by LC–ESI–MS/MS. In a validation cohort, we confirmed three of them (FGA, AHSG and APOA-I) via ELISA. Our data suggested a relative high diagnostic accuracy of the above three biomarkers in GC patients.

Alpha 2-HS glycoprotein (AHSG), also known as Fetuin-A, is synthesized by hepatocytes and secreted into the circulatory system [19]. AHSG is a multifunctional glycoprotein involved in numerous normal and pathological processes such as brain development, bone metabolism regulation, insulin resistance, migration and invasion in human colorectal cancer [20]. In addition, AHSG had also been described as a potential diagnostic biomarker for cancer patients. Yi et al. [21] reported that serum AHSG could be used in breast cancer diagnosis. Chen et al. [22] also suggested that the high serum level of AHSG could be a potential biomarker for patients with pancreatic cancer. Moreover, the diagnostic value of AHSG had also been reported in hepatocellular carcinoma [23], glioblastoma [24], esophageal squamous cell carcinoma [25], and prostate cancer [26]. In our study, we reported for the first time that the serum level of AHSG was higher in GC patients than in healthy controls. Our data suggested that serum AHSG could be a potential biomarker for GC patients, especially for those patients with late-stage GC.

Fibrinogen alpha chain (FGA) is one of three types of non-identical polypeptide chains consisting of fibrinogen, which is a blood-borne glycoprotein. Comprehensive research indicated that the serum level of FGA was up-regulated in many malignant neoplasms, such as breast cancer, nasopharyngeal carcinoma, acute lymphocytic leukemia, esophageal squamous cell carcinoma and renal cell cancer [27–29]. Our data based on mass spectrometry revealed that 5 out of 12 candidate peptides were identified as FGA. Moreover, in the ELISA-based validation cohort, the serum level of FGA was significantly higher in GC patients than in healthy controls, and serum FGA showed favorable diagnostic accuracy in patients with both early- and late-stage GC. Our finding is consistent with those of previous studies by Liu et al. [30] and Wu et al. [31]. The high serum level of FGA in cancer patients may be due to the release of pro-coagulant factors from endothelial cells and platelets, the latter of which were stimulated by cancer cells [32, 33]. In addition, some studies also reported that FGA was involved in tumor progression and metastasis [34], which also suggested that serum FGA could be a potential tumor-associated biomarker to improve the sensitivity of the diagnosis of GC.

Recently, the relationship between apolipoprotein and cancer has been highlighted. Signe Borgquist et al. reported that overall cancer risk is associated with the circulatory content of apolipoproteins in males [35]. Recent studies have also revealed that some apolipoproteins, such as APOC-I [36] and APOC-III [37], were overexpressed in serum samples derived from GC patients. APOA-I, which is produced in the liver and intestine [38], is a primary structural and functional portion of high-density lipoprotein and plays an indispensable role in cholesterol transportation and metabolism homeostasis [39]. Due to its intricate biological functions, APOA-I has been reported to be involved in various pathological processes, such as cardiovascular disease, myeloproliferative disorders and Alzheimer’s disease [40]. Moreover, it was observed that APOA-I could be secreted by cancer cells [41, 42], suggesting the tight association between APOA-I and cancer. In the clinic, the diagnostic value of APOA-I as a potential tumor biomarker was also reported in multiple malignancies, such as breast cancer, lung cancer, ovarian cancer, bladder cancer and cholangiocarcinoma [40]. In our study, APOA-I was shown to be up-regulated in GC patients, especially in those with early-stage GC. Moreover, the level of APOA-I was significantly decreased after gastrectomy, suggesting that this biomarker reflects tumor burden. Our findings are consistent with those of a previous study [43] that found an association between the circulating level of APOA-I and tumor burden in a mouse model. However, in a previous study by Muntoni et al. [44], the level of serum APOA-I in female GC patients was decreased compared with healthy controls, and there was no significant difference between male GC patients and healthy controls. The inconsistent results in our study and Muntoni’s study may be due to the following reasons. First, the apolipoprotein isoforms and the level of lipoprotein were reported to be highly variable and ethnicity-specific [45]. Second, the apolipoproteins were unstable, resulting in the generation of some vulnerable protein fragments during prolonged storage [46]. In Muntoni’s study, the collection period of serum samples was more than 10 years (1984–1998). In contrast, our samples were consecutively collected within 10 months, which might reduce the degradation of serum proteins. Finally, the different methods of sample preparation and detection in the two studies could also contribute to the discrepancy, which needs to be validated by a large-sample size and multi-center cohort in the future.

## Conclusion

Twelve peptides were identified as candidate biomarkers for GC patients by high-throughput proteomics. Three of them (FGA, AHSG and APOA-I) were validated in a larger cohort with high diagnostic accuracies, which suggested that FGA, AHSG and APOA-I might be developed as potential diagnostic biomarkers. Meanwhile, all proteins identified are highly abundant serum proteins and the presence of different elevated levels of the proteins in the other cohort, which suggested these candidate biomarkers could probably be secondary biomarkers. Nevertheless, they are still potentially valuable to improve the sensitivity and specificity of the diagnosis of GC. Further reliable validation with bigger sample cohorts is still needed for these three potential GC biomarkers, which might be valuable for the clinical diagnosis of GC in the future. Moreover, further studies will focus on validating the diagnostic accuracies of another four non-validated potential biomarkers (HBB, TXNRD1, CAKP5, GSPT2) and on analyzing the relationships between the expressed levels of those biomarkers and the prognosis of GC patients.

## Additional file

**Additional file 1:** Supplementary material figures S1–S10.

## Abbreviations

GC: gastric cancer
CRC: colorectal cancer
HCC: hepatocellular carcinoma
MB-IMAC-Cu: magnetic beads based immobilized metal-ion affinity chromatography
MALDI-TOF/MS: matrix-assisted laser desorption/ionization time-of-flight mass spectrometry
CEA: carcino-embryonic antigen
CA19-9: carbohydrate antigen 19-9
CA72-4: carbohydrate antigen 72-4
AHSG: alpha 2-HS glycoprotein
APOA-I: apolipoprotein A-I
TXNRD1: isoform 5 of thioredoxin reductase 1, cytoplasmic
HBB: hemoglobin subunit beta
GSPT2: eukaryotic peptide chain release factor GTP-binding subunit ERF3B
CAKP5: cytoskeleton-associated protein 5
ELISA: enzyme-linked immunosorbent assay
