# Supplementary material for: Identification of serum proteins AHSG, FGA and APOA-I as diagnostic biomarkers for gastric cancer
Source: Clin Proteomics. 2018 Apr 30;15:18. doi: 10.1186/s12014-018-9194-0 (PMC5925839; doi:10.1186/s12014-018-9194-0)
Supplement: Supplementary file 1 — Additional file 1: Supplementary material figures S1–S10. [file 12014_2018_9194_MOESM1_ESM.docx]

**Figure S1 MS/MS identification of peak 1521.93 Da**


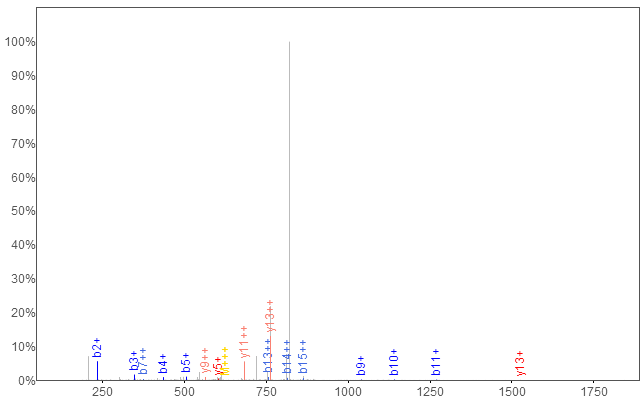


|  | **b+** | **b2+** | **#** | **Seq** | **#** | **y+** | **y2+** |  |
| --- | --- | --- | --- | --- | --- | --- | --- | --- |
|  | 88.0393 | 44.5233 | 1 | **S** | 16 |  |  |  |
|  | **235.1077** | 118.0575 | 2 | **F** | 15 | 1784.9429 | 892.9751 |  |
|  | **348.1918** | 174.5995 | 3 | **L** | 14 | 1637.8745 | 819.4409 |  |
|  | **435.2238** | 218.1155 | 4 | **S** | 13 | **1524.7904** | **762.8988** |  |
|  | **506.2609** | 253.6341 | 5 | **A** | 12 | 1437.7584 | 719.3828 |  |
|  | 619.3450 | 310.1761 | 6 | **L** | 11 | 1366.7213 | **683.8643** |  |
|  | 748.3876 | **374.6974** | 7 | **E** | 10 | 1253.6372 | 627.3222 |  |
|  | 877.4302 | 439.2187 | 8 | **E** | 9 | 1124.5946 | **562.8009** |  |
|  | **1040.4935** | 520.7504 | 9 | **Y** | 8 | 995.5520 | 498.2796 |  |
|  | **1141.5412** | 571.2742 | 10 | **T** | 7 | 832.4887 | 416.7480 |  |
|  | **1269.6361** | 635.3217 | 11 | **K** | 6 | 731.4410 | 366.2241 |  |
|  | 1397.7311 | 699.3692 | 12 | **K** | 5 | **603.3461** | 302.1767 |  |

|  | 1510.8152 | **755.9112** | 13 | **L** | 4 | 475.2511 | 238.1292 |  |
| --- | --- | --- | --- | --- | --- | --- | --- | --- |
|  | 1624.8581 | **812.9327** | 14 | **N** | 3 | 362.1670 | 181.5872 |  |
|  | 1725.9058 | **863.4565** | 15 | **T** | 2 | 248.1241 | 124.5657 |  |
|  |  |  | 16 | **Q** | 1 | 147.0764 | 74.0418 |  |

**Figure S2 MS/MS identification of peak 1265.49 Da**


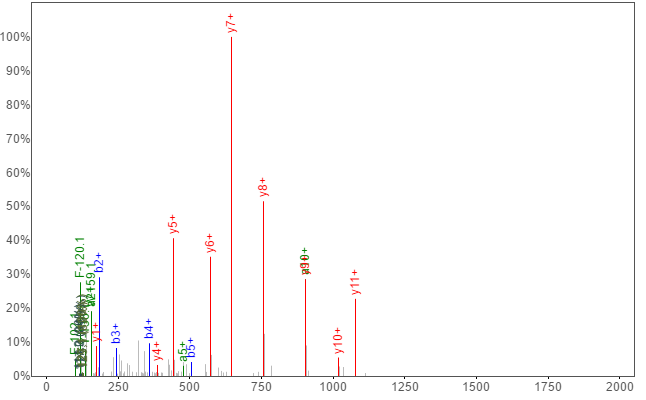


| **a+** | **b+** | **b2+** | **#** | **Seq** | **#** | **y+** | **y2+** |
| --- | --- | --- | --- | --- | --- | --- | --- |
| 30.0338 | 58.0287 | 29.5180 | 1 | **G** | 13 |  |  |
| **159.0764** | **187.0713** | 94.0393 | 2 | **E** | 12 | 1206.5749 | 603.7911 |
| 216.0979 | **244.0928** | 122.5500 | 3 | **G** | 11 | **1077.5323** | 539.2698 |
| 331.1248 | **359.1197** | 180.0635 | 4 | **D** | 10 | **1020.5109** | 510.7591 |
| **478.1932** | **506.1882** | 253.5977 | 5 | **F** | 9 | **905.4839** | 453.2456 |
| 591.2773 | 619.2722 | 310.1397 | 6 | **L** | 8 | **758.4155** | 379.7114 |
| 662.3144 | 690.3093 | 345.6583 | 7 | **A** | 7 | **645.3315** | 323.1694 |
| 791.3570 | 819.3519 | 410.1796 | 8 | **E** | 6 | **574.2944** | 287.6508 |
| 848.3785 | 876.3734 | 438.6903 | 9 | **G** | 5 | **445.2518** | 223.1295 |
| **905.3999** | 933.3949 | 467.2011 | 10 | **G** | 4 | **388.2303** | 194.6188 |
| 962.4214 | 990.4163 | 495.7118 | 11 | **G** | 3 | 331.2088 | 166.1081 |
| 1061.4898 | 1089.4847 | 545.2460 | 12 | **V** | 2 | 274.1874 | 137.5973 |
|  |  |  | 13 | **R** | 1 | **175.1190** | 88.0631 |

**Figure S3 MS/MS identification of peak 1352.84 Da**


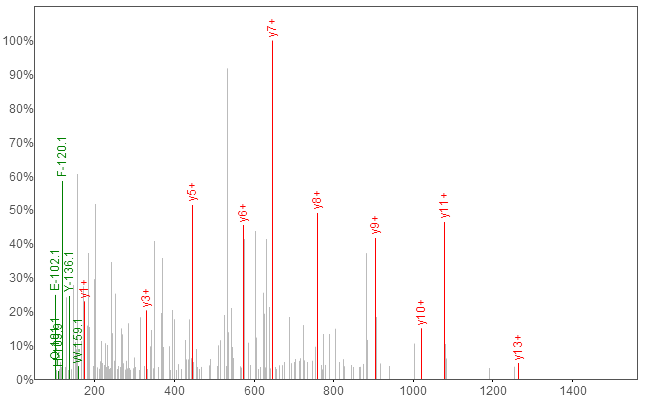


|  | **b+** | **#** | **Seq** | **#** | **y+** |  |
| --- | --- | --- | --- | --- | --- | --- |
|  | 116.0342 | 1 | **D** | 15 |  |  |
|  | 283.0326 | 2 | **S** | 14 | 1430.5948 |  |
|  | 340.0540 | 3 | **G** | 13 | **1263.5964** |  |
|  | 469.0966 | 4 | **E** | 12 | 1206.5749 |  |
|  | 526.1181 | 5 | **G** | 11 | **1077.5323** |  |
|  | 641.1450 | 6 | **D** | 10 | **1020.5109** |  |
|  | 788.2135 | 7 | **F** | 9 | **905.4839** |  |
|  | 901.2975 | 8 | **L** | 8 | **758.4155** |  |
|  | 972.3346 | 9 | **A** | 7 | **645.3315** |  |
|  | 1101.3772 | 10 | **E** | 6 | **574.2944** |  |
|  | 1158.3987 | 11 | **G** | 5 | **445.2518** |  |
|  | 1215.4202 | 12 | **G** | 4 | 388.2303 |  |
|  | 1272.4416 | 13 | **G** | 3 | **331.2088** |  |
|  | 1371.5100 | 14 | **V** | 2 | 274.1874 |  |

|  |  | 15 | **R** | 1 | **175.1190** |  |
| --- | --- | --- | --- | --- | --- | --- |

**Figure S4 MS/MS identification of peak 1575.90 Da**


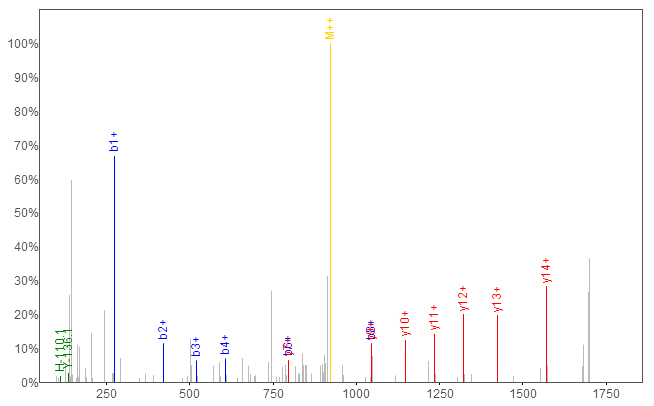


|  | **b+** | **#** | **Seq** | **#** | **y+** |  |
| --- | --- | --- | --- | --- | --- | --- |
|  | **273.1679** | 1 | **Q** | 15 |  |  |
|  | **420.2363** | 2 | **F** | 14 | **1569.6816** |  |
|  | **521.2840** | 3 | **T** | 13 | **1422.6132** |  |
|  | **608.3160** | 4 | **S** | 12 | **1321.5655** |  |
|  | 695.3481 | 5 | **S** | 11 | **1234.5335** |  |
|  | **796.3957** | 6 | **T** | 10 | **1147.5014** |  |
|  | 883.4278 | 7 | **S** | 9 | **1046.4538** |  |
|  | **1046.4911** | 8 | **Y** | 8 | 959.4217 |  |
|  | 1160.5340 | 9 | **N** | 7 | **796.3584** |  |

|  | 1316.6351 | 10 | **R** | 6 | 682.3155 |  |
| --- | --- | --- | --- | --- | --- | --- |
|  | 1373.6566 | 11 | **G** | 5 | 526.2144 |  |
|  | 1488.6835 | 12 | **D** | 4 | 469.1929 |  |
|  | 1575.7156 | 13 | **S** | 3 | 354.1660 |  |
|  | 1676.7633 | 14 | **T** | 2 | 267.1339 |  |
|  |  | 15 | **F** | 1 | 166.0863 |  |

**Figure S5 MS/MS identification of peak 2663.12 Da**


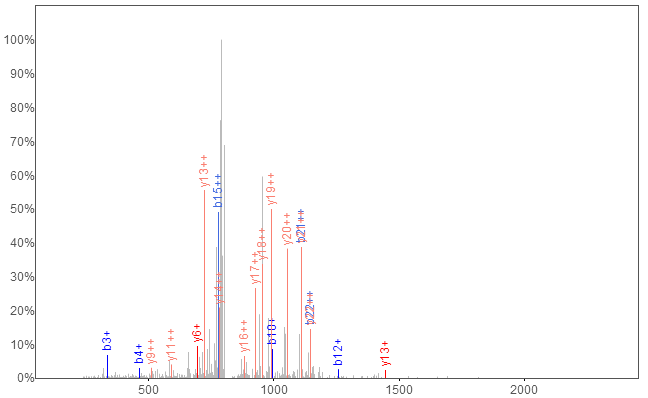


|  | **b+** | **b2+** | **#** | **Seq** | **#** | **y+** | **y2+** |  |
| --- | --- | --- | --- | --- | --- | --- | --- | --- |
|  | 148.0427 | 74.5250 | 1 | **M** | 23 |  |  |  |
|  | 219.0798 | 110.0435 | 2 | **A** | 22 | 2291.0395 | **1146.0234** |  |
|  | **334.1067** | 167.5570 | 3 | **D** | 21 | 2220.0024 | **1110.5049** |  |
|  | **463.1493** | 232.0783 | 4 | **E** | 20 | 2104.9755 | **1052.9914** |  |
|  | 534.1864 | 267.5969 | 5 | **A** | 19 | 1975.9329 | **988.4701** |  |
|  | 591.2079 | 296.1076 | 6 | **G** | 18 | 1904.8958 | **952.9515** |  |
|  | 678.2399 | 339.6236 | 7 | **S** | 17 | 1847.8743 | **924.4408** |  |

|  | 807.2825 | 404.1449 | 8 | **E** | 16 | 1760.8423 | **880.9248** |  |
| --- | --- | --- | --- | --- | --- | --- | --- | --- |
|  | 878.3196 | 439.6635 | 9 | **A** | 15 | 1631.7997 | 816.4035 |  |
|  | **993.3466** | 497.1769 | 10 | **D** | 14 | 1560.7626 | **780.8849** |  |
|  | 1130.4055 | 565.7064 | 11 | **H** | 13 | **1445.7356** | **723.3715** |  |
|  | **1259.4481** | 630.2277 | 12 | **E** | 12 | 1308.6767 | 654.8420 |  |
|  | 1316.4696 | 658.7384 | 13 | **G** | 11 | 1179.6341 | **590.3207** |  |
|  | 1417.5172 | 709.2623 | 14 | **T** | 10 | 1122.6127 | 561.8100 |  |
|  | 1554.5761 | **777.7917** | 15 | **H** | 9 | 1021.5650 | **511.2861** |  |
|  | 1641.6082 | 821.3077 | 16 | **S** | 8 | 884.5061 | 442.7567 |  |
|  | 1742.6558 | 871.8316 | 17 | **T** | 7 | 797.4740 | 399.2407 |  |
|  | 1870.7508 | 935.8790 | 18 | **K** | 6 | **696.4264** | 348.7168 |  |
|  | 2026.8519 | 1013.9296 | 19 | **R** | 5 | 568.3314 | 284.6693 |  |
|  | 2083.8734 | 1042.4403 | 20 | **G** | 4 | 412.2303 | 206.6188 |  |
|  | 2220.9323 | **1110.9698** | 21 | **H** | 3 | 355.2088 | 178.1081 |  |
|  | 2291.9694 | **1146.4883** | 22 | **A** | 2 | 218.1499 | 109.5786 |  |
|  |  |  | 23 | **K** | 1 | 147.1128 | 74.0600 |  |

**Figure S6 MS/MS identification of peak 2716.91 Da**


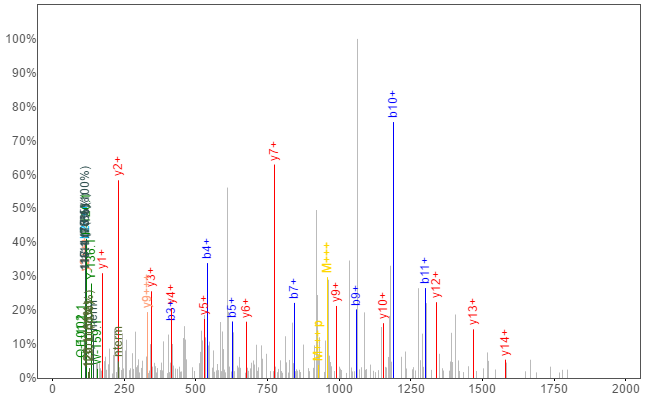


| **a+** | **b+** | **b2+** | **b3+** | **#** | **Seq** | **#** | **y+** | **y2+** | **y3+** |
| --- | --- | --- | --- | --- | --- | --- | --- | --- | --- |
| 216.1828 | 244.1778 | 122.5925 | 82.0641 | 1 | **V** | 25 |  |  |  |
| 315.2513 | 343.2462 | 172.1267 | **115.0869** | 2 | **V** | 24 | 2640.2821 | 1320.6447 | 880.7656 |
| 386.2884 | **414.2833** | 207.6453 | 138.7659 | 3 | **A** | 23 | 2541.2137 | 1271.1105 | 847.7428 |
| 514.3469 | **542.3419** | 271.6746 | 181.4521 | 4 | **Q** | 22 | 2470.1766 | 1235.5919 | 824.0637 |
| 601.3790 | **629.3739** | 315.1906 | 210.4628 | 5 | **S** | 21 | 2342.1180 | 1171.5627 | 781.3775 |
| 702.4267 | 730.4216 | 365.7144 | 244.1454 | 6 | **T** | 20 | 2255.0860 | 1128.0466 | 752.3669 |
| 816.4696 | **844.4645** | 422.7359 | 282.1597 | 7 | **N** | 19 | 2154.0383 | 1077.5228 | 718.6843 |
| 903.5016 | 931.4965 | 466.2519 | 311.1704 | 8 | **S** | 18 | 2039.9954 | 1020.5013 | 680.6700 |
| 1032.5442 | **1060.5391** | 530.7732 | 354.1846 | 9 | **E** | 17 | 1952.9634 | 976.9853 | 651.6593 |
| 1161.5868 | **1189.5817** | 595.2945 | 397.1988 | 10 | **E** | 16 | 1823.9208 | 912.4640 | 608.6451 |
| 1274.6709 | **1302.6658** | 651.8365 | 434.8934 | 11 | **I** | 15 | 1694.8782 | 847.9427 | 565.6309 |
| 1387.7549 | 1415.7498 | 708.3786 | 472.5881 | 12 | **I** | 14 | **1581.7941** | 791.4007 | 527.9362 |
| 1516.7975 | 1544.7924 | 772.8999 | 515.6023 | 13 | **E** | 13 | **1468.7101** | 734.8587 | 490.2415 |
| 1573.8190 | 1601.8139 | 801.4106 | 534.6095 | 14 | **G** | 12 | **1339.6675** | 670.3374 | 447.2273 |
| 1702.8616 | 1730.8565 | 865.9319 | 577.6237 | 15 | **E** | 11 | 1282.6460 | 641.8266 | 428.2202 |
| 1865.9249 | 1893.9198 | 947.4635 | 631.9781 | 16 | **Y** | 10 | **1153.6034** | 577.3053 | 385.2060 |
| 1979.9678 | 2007.9627 | 1004.4850 | 669.9924 | 17 | **N** | 9 | **990.5401** | 495.7737 | **330.8515** |
| 2081.0155 | 2109.0104 | 1055.0088 | 703.6750 | 18 | **T** | 8 | 876.4971 | 438.7522 | 292.8372 |
| 2180.0839 | 2208.0788 | 1104.5431 | 736.6978 | 19 | **V** | 7 | **775.4495** | 388.2284 | 259.1547 |
| 2327.1193 | 2355.1142 | 1178.0608 | 785.7096 | 20 | **M** | 6 | **676.3811** | 338.6942 | 226.1319 |
| 2440.2034 | 2468.1983 | 1234.6028 | 823.4043 | 21 | **L** | 5 | **529.3457** | 265.1765 | 177.1201 |
| 2511.2405 | 2539.2354 | 1270.1213 | 847.0833 | 22 | **A** | 4 | **416.2616** | 208.6344 | 139.4254 |
| 2624.3246 | 2652.3195 | 1326.6634 | 884.7780 | 23 | **I** | 3 | **345.2245** | 173.1159 | **115.7463** |
| 2681.3460 | 2709.3409 | 1355.1741 | 903.7852 | 24 | **G** | 2 | **232.1404** | **116.5738** | 78.0517 |
|  |  |  |  | 25 | **R** | 1 | **175.1190** | 88.0631 | 59.0445 |

**Figure S7 MS/MS identification of peak 4213.82Da**


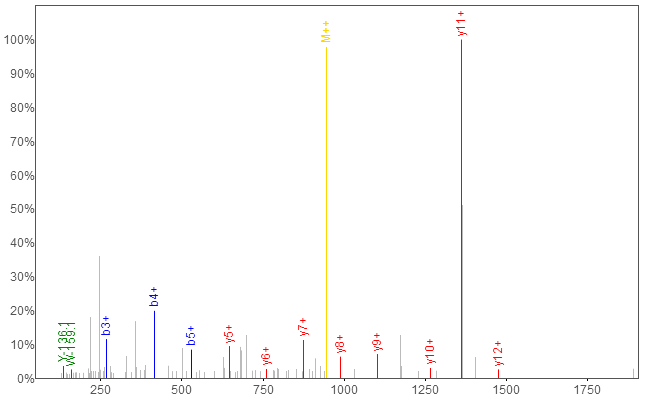


| **b+** | **#** | **Seq** | **#** | **y+** |
| --- | --- | --- | --- | --- |
| 58.0287 | 1 | **G** | 16 |  |
| 171.1128 | 2 | **L** | 15 | 1832.9694 |
| **268.1656** | 3 | **P** | 14 | 1719.8853 |
| **415.2340** | 4 | **F** | 13 | 1622.8326 |
| **528.3180** | 5 | **I** | 12 | **1475.7641** |
| 625.3708 | 6 | **P** | 11 | **1362.6801** |
| 788.4341 | 7 | **Y** | 10 | **1265.6273** |
| 901.5182 | 8 | **L** | 9 | **1102.5640** |
| 1016.5451 | 9 | **D** | 8 | **989.4799** |
| 1130.5881 | 10 | **N** | 7 | **874.4530** |
| 1243.6721 | 11 | **L** | 6 | **760.4100** |
| 1340.7249 | 12 | **P** | 5 | **647.3260** |
| 1454.7678 | 13 | **N** | 4 | 550.2732 |
| 1601.8362 | 14 | **F** | 3 | 436.2303 |
| 1715.8792 | 15 | **N** | 2 | 289.1619 |
|  | 16 | **R** | 1 | 175.1190 |


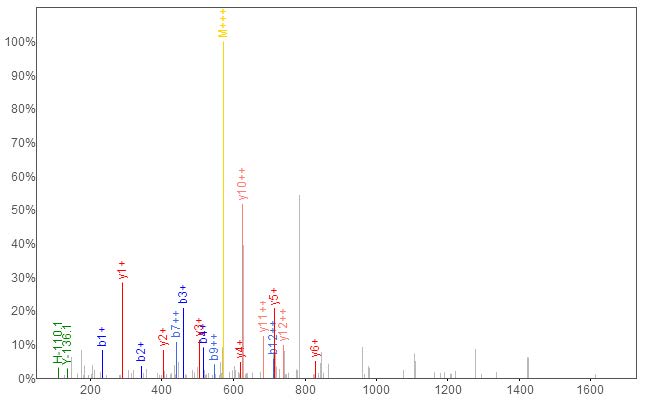


| **b+** | **b2+** | **#** | **Seq** | **#** | **y+** | **y2+** |
| --- | --- | --- | --- | --- | --- | --- |
| **232.1414** | 116.5743 | 1 | **S** | 13 |  |  |
| **345.2254** | 173.1164 | 2 | **I** | 12 | 1479.9015 | **740.4544** |
| **460.2524** | 230.6298 | 3 | **D** | 11 | 1366.8175 | **683.9124** |
| **517.2738** | 259.1406 | 4 | **G** | 10 | 1251.7905 | **626.3989** |
| 614.3266 | 307.6669 | 5 | **P** | 9 | 1194.7691 | 597.8882 |
| 727.4107 | 364.2090 | 6 | **I** | 8 | 1097.7163 | 549.3618 |
| 883.5118 | **442.2595** | 7 | **R** | 7 | 984.6322 | 492.8198 |
| 996.5958 | 498.8016 | 8 | **L** | 6 | **828.5311** | 414.7692 |
| 1093.6486 | **547.3279** | 9 | **P** | 5 | **715.4471** | 358.2272 |
| 1206.7327 | 603.8700 | 10 | **I** | 4 | **618.3943** | 309.7008 |
| 1305.8011 | 653.4042 | 11 | **V** | 3 | **505.3102** | 253.1587 |
| 1420.8280 | **710.9177** | 12 | **D** | 2 | **406.2418** | 203.6245 |
|  |  | 13 | **K** | 1 | **291.2149** | 146.1111 |

**Figure S8 MS/MS identification of peak 1506.07Da**


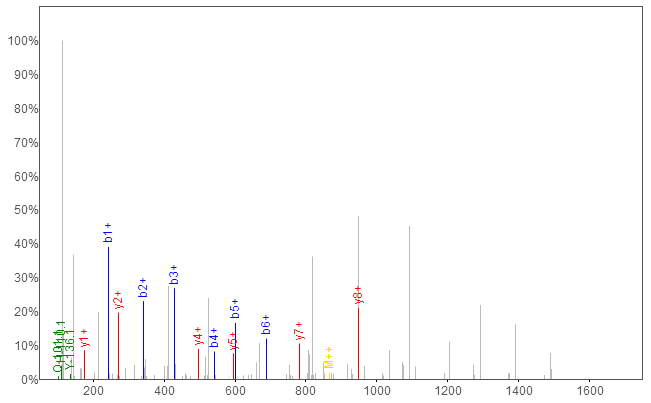


| **b+** | **#** | **Seq** | **#** | **y+** |
| --- | --- | --- | --- | --- |
| **244.1778** | 1 | **V** | 15 |  |
| **343.2462** | 2 | **V** | 14 | 1488.6842 |
| **430.2782** | 3 | **S** | 13 | 1389.6158 |
| **543.3623** | 4 | **L** | 12 | 1302.5838 |
| **600.3837** | 5 | **G** | 11 | 1189.4997 |
| **687.4158** | 6 | **S** | 10 | 1132.4783 |
| 784.4685 | 7 | **P** | 9 | 1045.4463 |
| 951.4669 | 8 | **S** | 8 | **948.3935** |
| 1008.4883 | 9 | **G** | 7 | **781.3951** |
| 1137.5309 | 10 | **E** | 6 | 724.3737 |
| 1236.5993 | 11 | **V** | 5 | **595.3311** |
| 1323.6314 | 12 | **S** | 4 | **496.2627** |
| 1460.6903 | 13 | **H** | 3 | 409.2306 |
| 1557.7430 | 14 | **P** | 2 | **272.1717** |
|  | 15 | **R** | 1 | **175.1190** |

**Figure S9 MS/MS identification of peak 1629.81 Da**


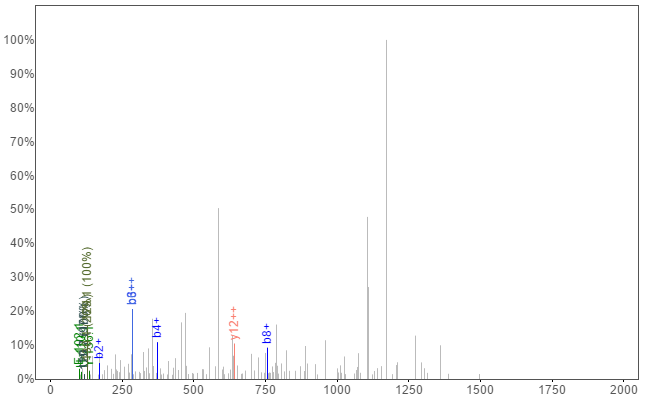


| **a+** | **b+** | **b2+** | **#** | **Seq** | **#** | **y+** | **y2+** |
| --- | --- | --- | --- | --- | --- | --- | --- |
| 30.0338 | 58.0287 | 29.5180 | 1 | **G** | 16 |  |  |
| 145.0608 | **173.0557** | 87.0315 | 2 | **D** | 15 | 1599.8449 | 800.4261 |
| 258.1448 | **286.1397** | 143.5735 | 3 | **L** | 14 | 1484.8180 | 742.9126 |
| 345.1769 | **373.1718** | 187.0895 | 4 | **S** | 13 | 1371.7339 | 686.3706 |
| 446.2245 | 474.2195 | 237.6134 | 5 | **T** | 12 | 1284.7019 | **642.8546** |
| 543.2773 | 571.2722 | **286.1397** | 6 | **P** | 11 | 1183.6542 | 592.3308 |
| 658.3042 | 686.2992 | 343.6532 | 7 | **D** | 10 | 1086.6015 | 543.8044 |
| 729.3414 | **757.3363** | 379.1718 | 8 | **A** | 9 | 971.5745 | 486.2909 |
| 828.4098 | 856.4047 | 428.7060 | 9 | **V** | 8 | 900.5374 | 450.7723 |
| 975.4452 | 1003.4401 | 502.2237 | 10 | **M** | 7 | 801.4690 | 401.2381 |
| 1032.4666 | 1060.4616 | 530.7344 | 11 | **G** | 6 | 654.4336 | 327.7204 |
| 1146.5096 | 1174.5045 | 587.7559 | 12 | **N** | 5 | 597.4121 | 299.2097 |
| 1243.5623 | 1271.5572 | 636.2823 | 13 | **P** | 4 | 483.3692 | 242.1882 |
| 1377.6774 | 1405.6723 | 703.3398 | 14 | **K** | 3 | 386.3164 | 193.6619 |
| 1476.7458 | 1504.7407 | 752.8740 | 15 | **V** | 2 | 252.2013 | 126.6043 |
|  |  |  | 16 | **K** | 1 | 153.1329 | 77.0701 |

**Figure S10 MS/MS identification of peak 2865.39 Da**


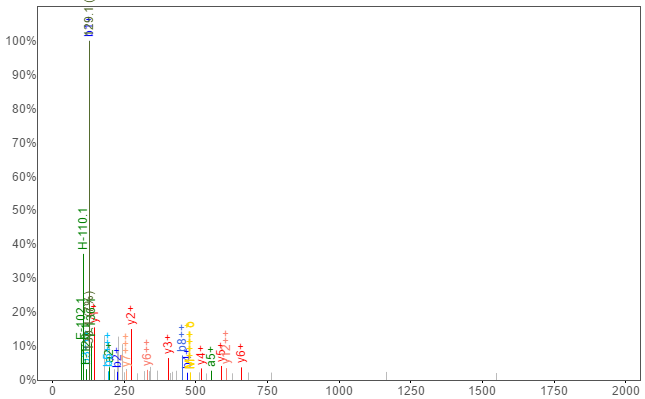


| **a+** | **b+** | **b2+** | **b3+** | **#** | **Seq** | **#** | **y+** | **y2+** | **y3+** |
| --- | --- | --- | --- | --- | --- | --- | --- | --- | --- |
| 101.1073 | **129.1022** | 65.0548 | 43.7056 | 1 | **K** | 18 |  |  |  |
| **200.1757** | **228.1707** | 114.5890 | 76.7284 | 2 | **V** | 17 | 1807.0436 | 904.0254 | 603.0194 |
| 329.2183 | 357.2132 | 179.1103 | **119.7426** | 3 | **E** | 16 | 1707.9752 | 854.4912 | 569.9966 |
| 442.3024 | **470.2973** | 235.6523 | 157.4373 | 4 | **L** | 15 | 1578.9326 | 789.9699 | 526.9824 |
| **555.3865** | 583.3814 | 292.1943 | **195.1320** | 5 | **I** | 14 | 1465.8485 | 733.4279 | 489.2877 |
| 692.4454 | 720.4403 | 360.7238 | 240.8183 | 6 | **H** | 13 | 1352.7645 | 676.8859 | 451.5930 |
| 749.4668 | 777.4617 | 389.2345 | 259.8254 | 7 | **G** | 12 | 1215.7056 | **608.3564** | 405.9067 |
| 877.5618 | 905.5567 | **453.2820** | 302.5238 | 8 | **K** | 11 | 1158.6841 | 579.8457 | 386.8996 |
| 1005.6568 | 1033.6517 | 517.3295 | 345.2221 | 9 | **K** | 10 | 1030.5891 | 515.7982 | 344.2012 |
| 1076.6939 | 1104.6888 | 552.8480 | 368.9011 | 10 | **A** | 9 | 902.4942 | 451.7507 | 301.5029 |
| 1133.7153 | 1161.7103 | 581.3588 | 387.9083 | 11 | **G** | 8 | 831.4571 | 416.2322 | 277.8239 |
| 1246.7994 | 1274.7943 | 637.9008 | 425.6030 | 12 | **L** | 7 | 774.4356 | 387.7214 | **258.8167** |
| 1317.8365 | 1345.8314 | 673.4194 | 449.2820 | 13 | **A** | 6 | **661.3515** | **331.1794** | 221.1220 |
| 1388.8736 | 1416.8685 | 708.9379 | 472.9610 | 14 | **A** | 5 | **590.3144** | 295.6608 | 197.4430 |
| 1503.9006 | 1531.8955 | 766.4514 | 511.3033 | 15 | **D** | 4 | **519.2773** | 260.1423 | 173.7640 |
| 1631.9955 | 1659.9905 | 830.4989 | 554.0017 | 16 | **K** | 3 | **404.2504** | 202.6288 | 135.4216 |
| 1760.0905 | 1788.0854 | 894.5463 | 596.7000 | 17 | **K** | 2 | **276.1554** | 138.5813 | 92.7233 |
|  |  |  |  | 18 | **E** | 1 | **148.0604** | 74.5339 | 50.0250 |


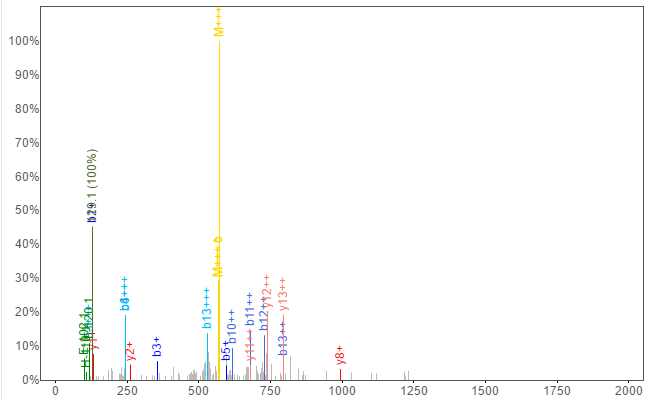


| **a+** | **b+** | **b2+** | **b3+** | **#** | **Seq** | **#** | **y+** | **y2+** | **y3+** |
| --- | --- | --- | --- | --- | --- | --- | --- | --- | --- |
| 101.1073 | **129.1022** | 65.0548 | 43.7056 | 1 | **K** | 14 |  |  |  |
| 214.1914 | 242.1863 | 121.5968 | 81.4003 | 2 | **L** | 13 | 1590.8407 | **795.9240** | 530.9518 |
| 329.2183 | **357.2132** | 179.1103 | **119.7426** | 3 | **D** | 12 | 1477.7567 | **739.3820** | 493.2571 |
| 457.3133 | 485.3082 | **243.1577** | 162.4409 | 4 | **K** | 11 | 1362.7297 | **681.8685** | 454.9148 |
| 570.3974 | **598.3923** | 299.6998 | 200.1356 | 5 | **I** | 10 | 1234.6348 | 617.8210 | 412.2164 |
| 698.4923 | 726.4872 | 363.7473 | **242.8339** | 6 | **K** | 9 | 1121.5507 | 561.2790 | 374.5218 |
| 827.5349 | 855.5298 | 428.2686 | 285.8481 | 7 | **E** | 8 | **993.4557** | 497.2315 | 331.8234 |
| 987.5656 | 1015.5605 | 508.2839 | 339.1917 | 8 | **C** | 7 | 864.4131 | 432.7102 | 288.8092 |
| 1074.5976 | 1102.5925 | 551.7999 | 368.2024 | 9 | **S** | 6 | 704.3825 | 352.6949 | 235.4657 |
| 1203.6402 | 1231.6351 | **616.3212** | 411.2166 | 10 | **E** | 5 | 617.3505 | 309.1789 | 206.4550 |
| 1331.7351 | 1359.7301 | **680.3687** | 453.9149 | 11 | **K** | 4 | 488.3079 | 244.6576 | 163.4408 |
| 1430.8036 | 1458.7985 | **729.9029** | 486.9377 | 12 | **V** | 3 | 360.2129 | 180.6101 | 120.7425 |
| 1559.8462 | 1587.8411 | **794.4242** | **529.9519** | 13 | **E** | 2 | **261.1445** | 131.0759 | 87.7197 |
|  |  |  |  | 14 | **L** | 1 | **132.1019** | 66.5546 | 44.7055 |
